# Deciphering N^6^-Methyladenosine-Related Genes Signature to Predict Survival in Lung Adenocarcinoma

**DOI:** 10.1155/2020/2514230

**Published:** 2020-02-29

**Authors:** Jie Zhu, Min Wang, Daixing Hu

**Affiliations:** ^1^Department of Intensive Care Unit, The People's Hospital of Tongliang District, Chongqing, China; ^2^Department of Respiratory and Geriatrics, Chongqing Public Health Medical Center, Chongqing, China; ^3^Department of Urology, The First Affiliated Hospital of Chongqing Medical University, Chongqing, China

## Abstract

Lung cancer is the most commonly diagnosed cancer and the leading cause of cancer-related death. Among these, lung adenocarcinoma (LUAD) accounts for most cases. Due to the improvement of precision medicine based on molecular characterization, the treatment of LUAD underwent significant changes. With these changes, the prognosis of LUAD becomes diverse. N^6^-methyladenosine (m^6^A) is the most predominant modification in mRNAs, which has been a research hotspot in the field of oncology. Nevertheless, little has been studied to reveal the correlations between the m^6^A-related genes and prognosis in LUAD. Thus, we conducted a comprehensive analysis of m^6^A-related gene expressions in LUAD patients based on The Cancer Genome Atlas (TCGA) database by revealing their relationship with prognosis. Different expressions of the m^6^A-related genes in tumor tissues and non-tumor tissues were confirmed. Furthermore, their relationship with prognosis was studied via Consensus Clustering Analysis, Principal Components Analysis (PCA), and Least Absolute Shrinkage and Selection Operator (LASSO) Regression. Based on the above analyses, a m^6^A-based signature to predict the overall survival (OS) in LUAD was successfully established. Among the 479 cases, we found that most of the m^6^A-related genes were differentially expressed between tumor and non-tumor tissues. Six genes, HNRNPC, METTL3, YTHDC2, KIAA1429, ALKBH5, and YTHDF1 were screened to build a risk scoring signature, which is strongly related to the clinical features pathological stages (*p* < 0.05), *M* stages (*p* < 0.05), *T* stages (*p* < 0.05), gender (*p* = 0.04), and survival outcome (*p* = 0.02). Multivariate Cox analysis indicated that risk value could be used as an independent prognostic factor, revealing that the m^6^A-related genes signature has great predictive value. Its efficacy was also validated by data from the Gene Expression Omnibus (GEO) database.

## 1. Introduction

Lung cancer is the most commonly diagnosed cancer and the leading cause of cancer-related death in men aged 75 years or older [1]. Even after radio and chemotherapy, the overall 5-year survival rate of lung cancer is less than 20% [2, 3]. Non-small-cell lung carcinomas (NSCLCs) account for almost 80% of cases and of which LUAD is the most common type [4]. The treatment of NSCLCs underwent significant changes in the last ten years by the improvement of precision medicine based on cellular and molecular characterization [5]. Identifying actionable genetic variants by molecular testing with small specimens obtained via minimally invasive technique has been a research hotspot at present.

**Figure 1:**
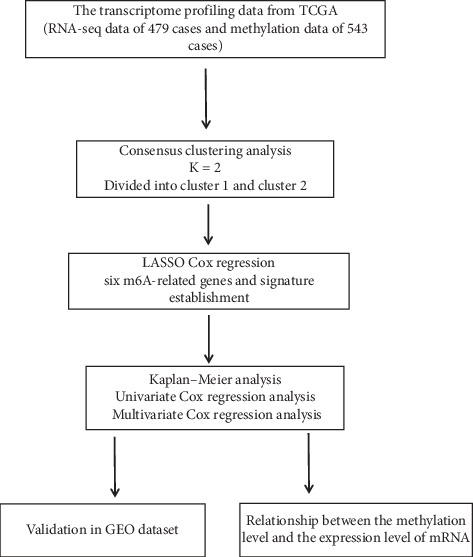
Workflow of the different analyses in the study.

RNAs are involved in a variety of cell processes that consist of more than 100 chemical modified nucleotides (including rRNA, tRNA, mRNA, snRNA, and others). Among them, mRNA plays a crucial role in the posttranscriptional regulation of gene expressions. The progress of adding a methyl(−CH3) group to a molecule is described as methylation, which can be observed on mRNA [6]. Of which, m^6^A is the most predominant modification that is presented in a DRACH motif (*D* = *A*, *G* or *U*; *R* = *A* or *G*; *H* = *A*, *C* or *U*) with identified sequence content [*G*/*A*/*U*][*G* > *A*]m^6^AC[*U* > *A* > *C*] [7, 8]. It has a significant impact on cancer cell fate by affecting the binding of the regulatory protein, changing the structure of RNAs, altering the maturity of the mRNA, and altering gene expression [9–12]. The process of methylation is controlled by the enzyme family called methyltransferases (writers), which catalyze the formation of the m^6^A level. The core components of encoding genes consist of methyltransferase-like 3 (METTL3), METTL14, and Wilms tumor 1 associated protein (WTAP), RNA-binding motif protein 15 (RBM15), zinc finger CCCH domain-containing protein 13 (ZC3H13), and vir-like m^6^A methyltransferase associated protein (VIRMA, also known as KIAA1429) [13–19]. It is coupled with a process of demethylation that can remove the methyl group to make the methylation reversible. It is achieved by another enzyme family called demethylases (erasers), encoded by fat mass and obesity-associated (FTO) and alkB homolog 5 (ALKBH5) [20–22]. Another family group of RNA-binding proteins (readers) that recognize these modifications also take part in carrying out different biological functions of mRNA. The encoding genes usually contain heterogeneous nuclear ribonucleoprotein C (HNRNPC) and the YTH domain families (YTHDC1, YTHDC2, YTHDF1, YTHDF2, and YTHDF3) [6, 23, 24]. While m^6^A is found to be associated with tumorigenesis in different types of cancers, little is known about the relationship between m^6^A-related genes and LUAD. Based on the transcription data and clinical data provided by TCGA database and the GEO database, we thus performed a comprehensive analysis to reveal the correlation between mRNA methylation and clinical features of patients with LUAD (Figure 1). In this study, we evaluated the alteration spectrum of fourteen m^6^A-related genes, as well as the association between the genetic alterations and clinical outcomes.

## 2. Methods

### 2.1. Samples and Data Extraction

The transcriptome profiling data, the methylation profiles, and the clinical information of LUAD patients were downloaded from TCGA database (https://portal.gdc.cancer.gov/) and the GEO database (https://www.ncbi.nlm.nih.gov/). In total, the RNA-seq data of 479 cases and the methylation data (beta values) of 543 cases were collected, which include lung tumor tissues and matched non-tumor tissues. We mainly focused on the expression of fourteen genes related to m^6^A (specifically, METTL3, METTL14, WTAP, RBM15, ZC3H13, KIAA1429, FTO, ALKBH5, HNRNPC, YTHDC1, YTHDC2, YTHDF1, YTHDF2, and YTHDF3) in these cases.

### 2.2. Statistical Analysis

These differentially expressed genes were screened using the package limma in *R* 3. 6. 0. The survival analysis was performed by the package survival. A risk scoring system was established via Consensus Clustering Analysis and LASSO regression. Consensus Clustering Analysis was used to classify LUAD patients into subtypes. It specified the optimal number of clusters to optimize the clustering model. PCA was used to evaluate the effectiveness of the Consensus Clustering Analysis classification. To get a more practical model, we used LASSO regression to screen the most powerful predictive prognostic genes with regression coefficients. The most important genes associated with m^6^A and the prognosis were selected to establish a formula, which was also evaluated to predict survival by the Kaplan-Meier method with hazard ratios (HR) calculated. Univariate Cox regression was used to analyze the clinical features and the risk score for association with OS. Multivariate Cox regression analysis indicated its independent prognostic value. The Chi-square test for parametric distributions or the Wilcoxon test for nonparametric distributions was used, respectively. We considered *p* < 0.05 significant for all comparisons.

## 3. Results

### 3.1. Consensus Clustering Analysis and PCA

**Figure 2:**
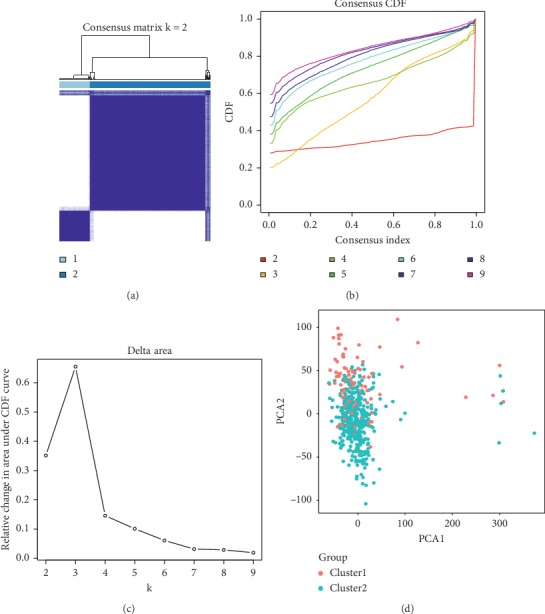
(a), (b), and (c) Consensus Clustering Analysis of the m^6^A-related genes, inferring the optimal number of clusters by taking the *K* value of 2. (d) Principal Components Analysis of the m^6^A-related genes. The genes of clusters 1 and 2 gather effectively, which indicates that the above classification based in Consensus Clustering Analysis is confirmed.

We could infer the optimal number of clusters by taking an appropriate *K* value. The best CDF value was obtained by taking the *k* value of 2 (Figures 2(a)–2(c)), suggesting that we could divide the patients into two groups. We applied PCA to confirm the effectiveness of Consensus Clustering Analysis. It showed two significantly different distribution patterns. The samples of cluster 1 and cluster 2 were distributed on the left side and the right side, respectively (Figure 2(d)), which indicated that our classification generated by Consensus Clustering Analysis was effective.

### 3.2. The Difference in m^6^A-Related Gene Expressions

**Figure 3:**
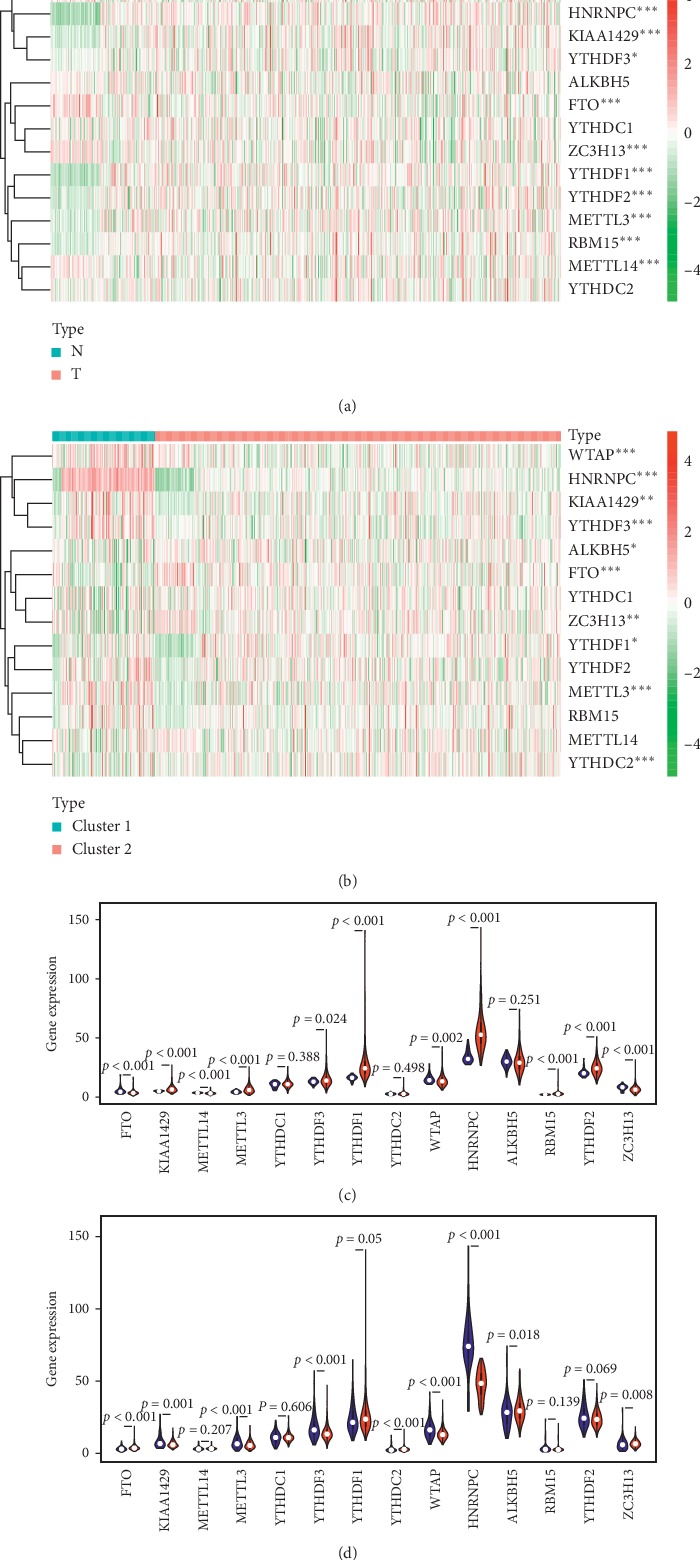
The profiles of the m^6^A-related genes for LUAD patients. (a) The difference in gene expressions between tumor tissues and non-tumor tissues. ^*∗*^represents *p* < 0.05, and ^*∗∗∗*^represents *p* < 0.001. *N* = non-tumor tissues, *T* = tumor tissues. (b) The difference of gene expressions between cluster 1 and cluster 2. ^*∗*^represents *p* < 0.05, and ^*∗∗∗*^represents *p* < 0.001. (c) The violin plot of the m^6^A-related gene expressions. Blue color represents non-tumor tissues, and the red color represents tumor tissues. (d) The violin plot of the m^6^A-related gene expressions. Blue color represents cluster 1, and the red color represents cluster 2.

**Figure 4:**
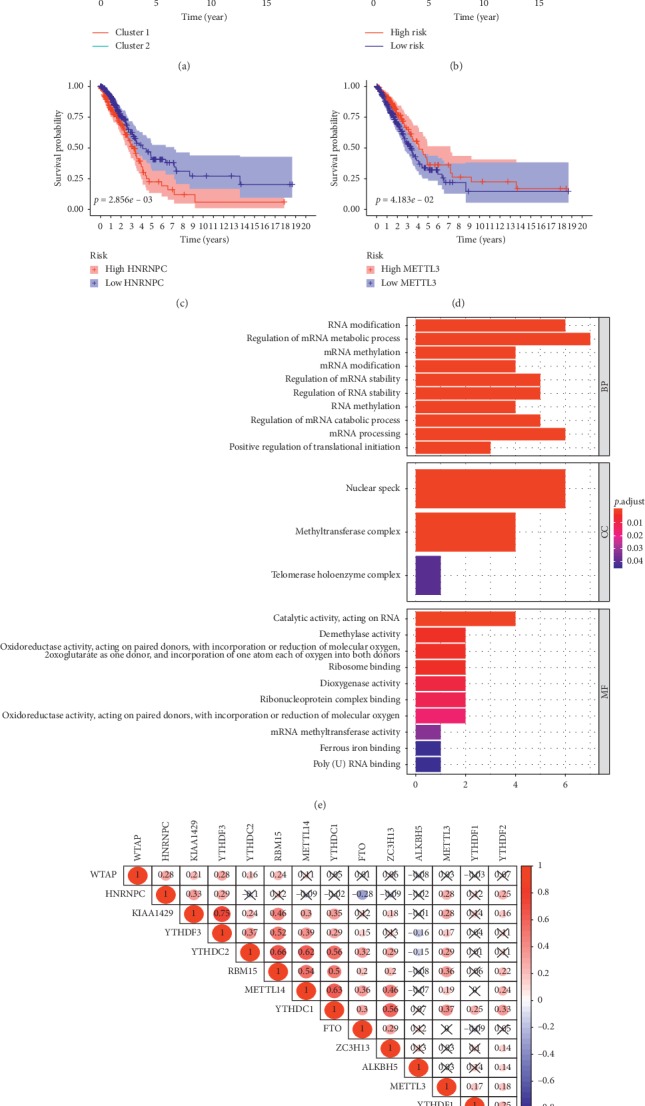
(a) The Kaplan-Meier survival curves indicate a significant difference between LUAD patients in cluster 1 and cluster 2. (b) The Kaplan-Meier survival curves indicate a significant difference between LUAD patients in high-risk and low-risk groups. (c) and (d) The Kaplan-Meier analysis shows that patients with highly expressed HNRNPC or METTLE3 have better survival rates. (e) 10 overrepresented biological processes in GO term functional enrichment were identified. (f) The correlation and correlation coefficients between m^6^A-related genes. Red color represents a positive correlation and blue color represents a negative correlation.

Among the 479 cases, 10 of the 14 genes were found to have differential expressions in the tumor and non-tumor tissues. The heatmap was conducted by the package pheatmap. Specifically, HNRNPC, YTHDF1, YTHDF2, METTL3, RBM15, YTHDF3, and KIAA1429 are highly expressed in tumor tissues. On the contrary, METTL14, ZC3H13, FTO, and WTAP are low-expressed in tumor tissues (Figures 3(a) and 3(c)). Enrichment analysis of the differential m^6^A-related genes offered a biological understanding. 10 overrepresented biological processes in gene ontology (GO) term functional enrichment (Figure 4(e)) were identified. Most biological processes were enriched in RNA modification, regulation of mRNA metabolic process, and mRNA methylation. Depending on the classification of Consensus Clustering Analysis, 10 of the 14 genes were found to have differential expressions. The heatmap and the violin plot of these genes were shown in Figures 3(b) and 3(d). In detail, HNRNPC, WTAP, YTHDF3, METTL3, and KIAA1429 are highly expressed in group cluster 1. Besides, FTO, YTHDC2, ZC3H13, ALKBH5, and YTHDF1 are high-expressed in cluster 2. The interaction and correlation between all genes were evaluated through the package corrplot, which suggested that the strongest positive correlation could be observed in YTHDF3 and KIAA1429, YTHDC2 and RBM15, and YTHDC2 and METTL14. Instead, the strongest negative correlations were shown between FTO and HNRNPC, YTHDF3 and ALKBH5, and YTHDC2 and ALKBH5 (Figure 4(f)). YTHDC2 seems to be involved as a hub gene in the interaction of m^6^A-related genes. Its interaction and coexpression with RBM15, METTL14, and ALKBH5 are also consistent with its reader role. ALKBH5 and FTO are prominent in the negative correlation of m^6^A-related genes, which is in line with their role as erasers of the m^6^A process. The most significant positive correlation exists in the writers and the readers' groups, which is consistent with the fact that the expression levels of KIAA1429, YTHDC2, and RBM15 are highly expressed in LUAD tumor tissues.

### 3.3. Functional Annotation of Differential m^6^A-Related Genes

#### 3.3.1. The Survival Analysis of Consensus Clustering Analysis

The survival curve was obtained via the Kaplan-Meier analysis (Figure 4(a)), which showed that the survival rate of cluster 2 patients (survival rate = 35.3%, 95% CI: 0.081 − 0.460) was better than that of cluster 1 patients (survival rate = 20.0%, 95% CI: 0.275 − 0.454). A significant statistical difference could be observed between them (*p* = 0.009), revealing that the typing based on m^6^A-related genes was significantly interrelated to the prognosis. These m^6^A-related genes can be used as possible factors to predict the prognosis of patients with LUAD.

#### 3.3.2. Identifying m^6^A-Related Gene Signature by LASSO Regression

To better predict the outcome of patients with LUAD, we conducted LASSO Cox regression to fourteen m^6^A-related genes (Supplement Figure 1). Finally, six genes (HNRNPC, METTL3, YTHDC2, KIAA1429, ALKBH5, and YTHDF1) were selected to establish the signature. The coefficients were obtained from the LASSO algorithm. All patients could be divided into the high-risk group and the low-risk group depending on their risk score, taking the average value as the cut-off point. The formula is generated as follows: Risk score = (0.008 × expression value of HNRNPC) + (−0.035 × expression value of METTL3) + (−0.009 × expression value of YTHDC2) + (0.033 × expression value of KIAA1429) + (−0.062 × expression value of ALKBH5) + (−0.062 × expression value of YTHDF1). Patients with higher risk scores tend to have worse prognosis (Figure 4(b)).

#### 3.3.3. The Relationship between the Signature Genes and the Different Clinicopathological Parameters

**Figure 5:**
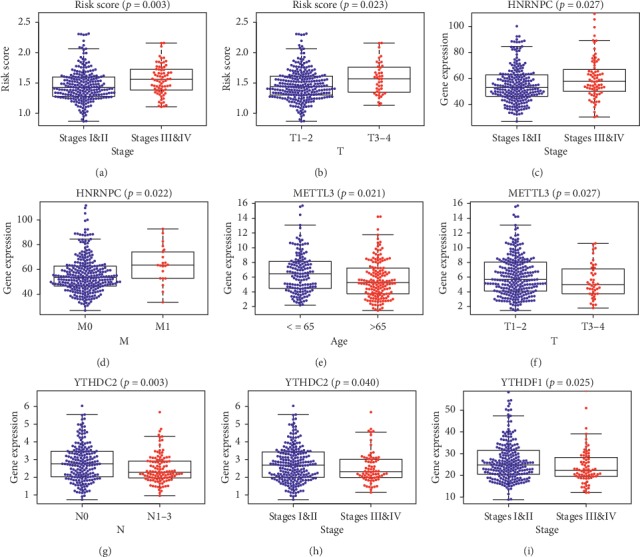
The relationship between the signature genes and the different clinicopathological parameters. (a) and (b) Patients with higher pathological stages tend to have higher risk scores. (c) and (d) Patients with higher pathological stages tend to have higher HNRNPC expressions. (e) and (f) Older patients and patients with higher pathological stages tend to have lower METTL3 expressions. (g) and (h) Patients with higher *N* stages and pathological stages tend to have lower YTHDC2 expressions. (i) Patients with higher pathological stages tend to have lower YTHDF1 expressions.

**Figure 6:**
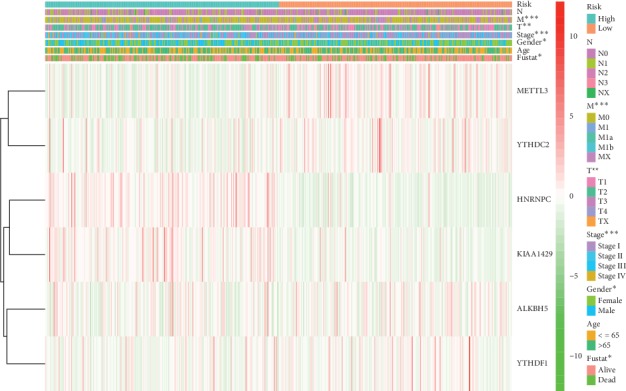
The heatmap of the relationship between the signature and clinical features via the Chi-square test. ^*∗*^represents *p* < 0.05, ^*∗∗*^represents *p* < 0.01, and ^*∗∗∗*^represents *p* < 0.001.

As shown in Figure 5, the correlation between risk scores and clinicopathological parameters was tested. The patients with higher pathological stages and *T* stages tend to score higher (Figures 5(a) and 5(b)). HNRNPC is highly expressed in patients with higher pathological stages and *M* stages (Figures 5(c) and 5(d)). The survival analysis also reveals that patients with lower expression levels of HNRNPC suggest a better prognosis (Figure 4(c)). METTL3 tends to be highly expressed in younger patients and patients with earlier *T* stages (Figures 5(e) and 5(f)). Instead, the Kaplan-Meier analysis also shows that highly expressed METTLE3 patients have better survival rates (Figure 4(d)). Patients with higher *N* stages and pathological stages seem to have lower YTHDC2 expressions than patients with lower*N* stages and pathological stages (Figures 5(g) and 5(h)). YTHDF1 shows a lower expression in the higher pathological stages (Figure 5(i)). However, we did not find a difference in the Kaplan-Meier analysis of YTHDC2 and YTHDF1. To further validate the clinical value of the signature, we evaluated the relationship between the signature and clinical features via the Chi-square test (Figure 6). The risk grouping was strongly related to pathological stages (*p* < 0.05), *M* stages (*p* < 0.05), *T* stages (*p* < 0.05), gender (*p* = 0.04), and survival outcome (*p* = 0.02).

#### 3.3.4. Determining the Survival Power and Predictive Ability of the m^6^A-Related Gene Signature

**Figure 7:**
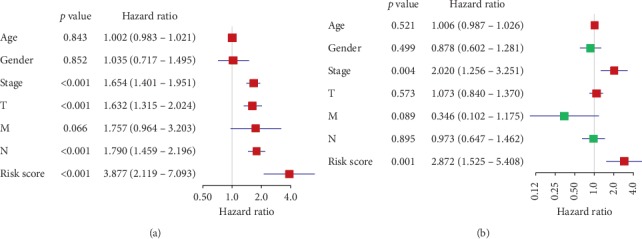
(a) Univariate Cox regression analysis of the risk score with OS as the dependent variable. (b) Multivariate Cox regression analysis of the risk score and clinical factors with OS as the dependent variables.

The Kaplan-Meier analysis (Figure 4(b)) of OS showed that low-risk group patients had significantly better OS than those in the high-risk group (*p* < 0.001). The 5-year OS rate of patients in the high-risk group was about 24.3%, while that of the patients in the low-risk group was 43.2%. Univariate Cox regression analysis (Figure 7(a)) showed that the risk score was negatively correlated with OS (high-risk group versus low-risk group, HR = 3.877, 95% CI: 2.119 − 7.093, *p* < 0.001) as well as the pathological stages, *T* stages, and *N* stages, suggesting a great predictive ability of the signature. Then, Multivariate Cox regression analysis (Figure 7(b)) was performed to show the prognostic power of the risk score. The result showed that the risk value played a role as an independent prognostic factor (high-risk group versus low-risk group, HR = 2.872, 95% CI: 1.525 − 5.408, *p* = 0.001).

#### 3.3.5. The Relationship between the Methylation Level and the Expression Level of mRNA for the Prognostic m^6^A-Related Genes

**Figure 8:**
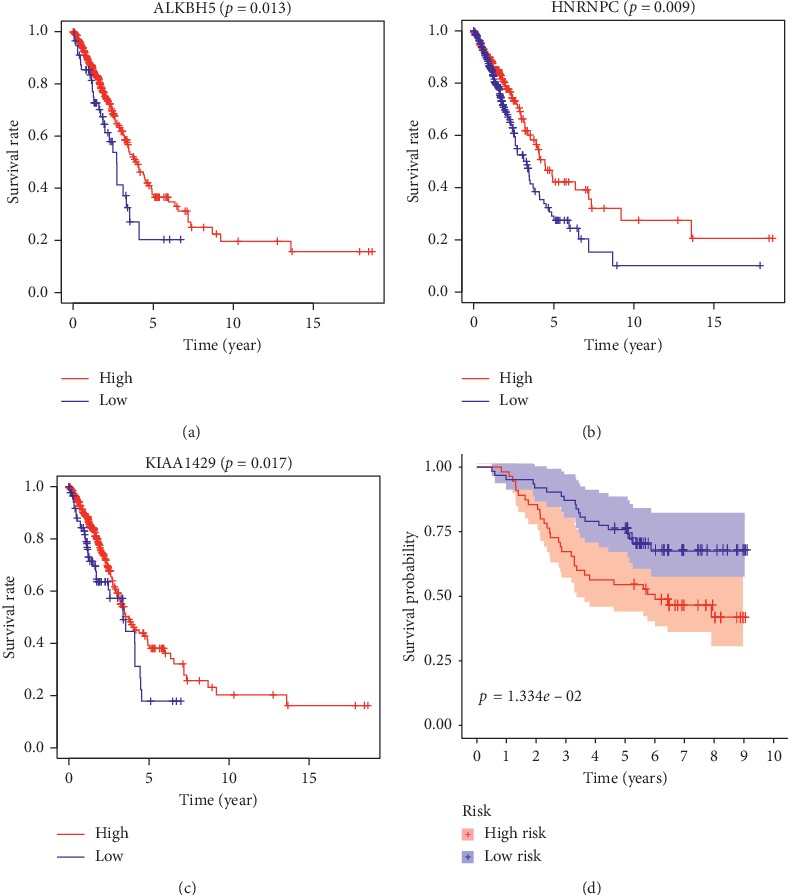
(a), (b), and (c) The Kaplan-Meier curves show that patients with hypermethylation levels of ALKBH5, HNRNPC, and KIAA1429 tend to have better OS. (d) The Kaplan-Meier analysis indicates the prognostic ability of m^6^A-related signature in the GEO dataset GSE13213.

The six prognostic m^6^A-related genes screened by LASSO regression were tested for the relevance between the methylation level and survival. Taking an optimal cut-off elaborated by an iterative approach (68.2%) stratifying patients into mPITX3 hyper-(mPITX3 high) and hypo-(pPITX3 low) cases, the Kaplan-Meier curves showed that patients with hypermethylation levels of ALKBH5, KIAA1429, and HNRNPC tended to have better OS (Figures 8(a), 8(b), and 8(c)). Considering that these genes are directly related to the prognosis of LUAD, the results suggest that the methylation levels of m^6^A-related genes may directly affect the prognosis of patients.

#### 3.3.6. Validation of m^6^A-Related Signature via an Independent Cohort

The GEO dataset GSE13213 was used as an independent external validation cohort. We calculated the risk score for each patient by the same formula. The patients were divided into high-risk and low-risk groups based on the median risk score. Through the result of the Kaplan-Meier analysis, the prognostic ability of our signature was confirmed again (Figure 8(d)). The high-risk patients had a lower OS than the low-risk patients (five-year survival rate = 54.5% versus 74.0%, *p* < 0.05). These validation experiments confirmed the valuable ability of our risk signature to predict the prognosis of LUAD patients.

## 4. Discussion

LUAD is often diagnosed at an advanced stage with a high mortality rate. Many studies suggested that the m^6^A process of mRNAs is linked to lung cancer, which makes m^6^A-related genes potential biomarkers for improving clinical management. Based on our study, m^6^A-related genes are differentially expressed in LUAD patients. The classification based on m^6^A-related genes was associated with the prognosis. We identified a signature composed of six m^6^A-related genes via different statistics and machine learning methods. To our knowledge, little has been revealed for these identified genes (HNRNPC, METTL3, YTHDC2, KIAA1429, ALKBH5, and YTHDF1) in the development and treatment of LUAD. According to the data of the Human Protein Atlas (HPA) database (based on TCGA database), none of the six prognostic-related genes is an independent prognostic predictor of lung cancer or LUAD.

HNRNPC relates to pre-mRNA processing and other aspects of mRNA metabolism. Proteins of HNRNPC are thought to be the potential m^6^A-selective binding proteins to affect mRNA localization and transport [8, 23]. Some studies have confirmed the interaction between HNRNPC and the urokinase receptor (uPAR) mRNA in lung-derived epithelial cells, which could contribute to the pathogenesis of lung neoplasia [25, 26]. In other cases, overexpressing HNRNPC in gastric cancer cells promotes chemoresistance [27]. METTL3 works as a writer to get control of the eukaryotic mRNA translation in the posttranscriptional methylation. It increases the translation of certain mRNA, including epidermal growth factor receptor (EGFR) and the Hippo pathway effector TAZ in human cancer cells [12]. Its role in the occurrence and development of lung cancer has been partly confirmed by some studies. The elevated expression of METTL3 in LUAD is thought to promote growth and invasion of cancer cells. The data of TCGA shows that the expression of METTL3 mRNA is significantly elevated in LUAD compared with the normal tissues [28]. Inhibiting the expression of METTL3 could reverse the positive effect of METTL3 on NSCLC progression [28]. Little information is revealed for the role of YTHDC2 in tumorigenesis. As one of the YTH domain families, YTHDC2 can affect different aspects of gene expression by recognizing and binding to RNAs containing m^6^As [30]. It contains additional RNA-binding and protein-protein interaction domains, which directs specific subsets of mRNAs for rapid expression and degradation, affecting the stability of its mRNA interaction partners [31]. Recent reports showed that there could be an oncogenic role of YTHDC2 in colon cancer cells and hepatocellular carcinoma cells [32, 33]. But based on the research of the TCGA database, YTHDC2 expression is positively associated with the prognosis of head and neck squamous cell carcinoma, indicating that it might also act as a tumor suppressor gene [34]. KIAA1429 encodes the largest known component of methyltransferase, which serves as a scaffold of the complex. It helps to bridge the catalytic core components of METTL3/METTL14/WTAP and RNA substrates, thus affecting the installation of m^6^A progress [35]. But the specific functions and mechanisms of KIAA1429 have not yet been fully elaborated. Some researchers have confirmed that KIAA1429 played its oncogenic role in breast cancer by regulating cyclin-dependent kinase 1 (CDK1) [36]. In hepatocellular carcinoma, KIAA1429 increased the m^6^A level of ID2 (a dominant-negative antagonist of transcription factors) mRNA, which subsequently reduced ID2 expression and promoted cell migration and invasion [14]. The protein of ALKBH5 is an m^6^A eraser protein. As an m^6^A eraser, it removes m^6^A from the targeting mRNAs by working closely with BCL-2. Specifically, ALKBH5 suppresses the BCL-2 expression, which was demonstrated to inhibit autophagy in cancer [37]. In breast cancer, ALKBH5 mediates the m^6^A-demethylation of mRNA, inducing the breast cancer stem cell phenotype [38, 39]. It was also reported to promote the malignant behavior of glioblastoma and epithelial ovarian cancer [40]. YTHDF1 functions as a reader of the m^6^A-modified mRNAs to facilitate translation initiation. The relationship between YTHDF1 and cancer has been reported in hepatocellular carcinoma [35, 41]. YTHDF1 has also been proved to be related to NSCLC cell proliferation. Its depletion renders cancerous cells resistant to cisplatin treatment. Low expression of YTHDF1 tended to a worse clinical outcome [42], which is consistent with our result that YTHDF1 showed lower expression in higher pathological stages.

In addition, we observed a positive or negative correlation between the expression of several genes in tumor tissues. It is worth noting that the expressions of METTL14 and METTL3 in tumor tissues are opposite. METTL3 is highly expressed in tumor tissues, while METTL14 expression is low. Given that both are methyltransferases, there seems to be a contradiction. The same situation also exists in METTL14 and FTO, both of which show low expression in tumor tissues. Given that one of them is methyltransferase, and the other is demethylase, the same low-expression trend seems contradictory.

Since the process of m^6^A often exhibits a reversible role in the mRNA expressions, we believe that m^6^A-related genes may have different functional patterns and functional networks when participating in malignancies. Therefore, there may be different expression patterns among m^6^A-related genes in LUAD. In previous studies, we know little about the interaction between m^6^A-related genes. Some studies have shown that METTL3, ALKBH5, YTHDC1, YTHDF1, YTHDF2, and HNRNPC are the main m^6^A-related genes in type II testicular germ cell tumors [29]. High ALKBH5 and HNRNPC, or low FTO and YTHDC2 staining intensities on the protein level, were observed as well as similar profiles on mRNA expression levels [29]. Lobo et al. stated that the expression of KIAA1429, YTHDF3, YTHDC1, METTL4, and ALKBH5 was significantly higher in seminomas than that in embryonal carcinomas, while METTL14 is expressed considerably lower [43]. It suggested that there may be specific interaction and expression patterns between m^6^A-related genes in particular tumors.

## 5. Conclusion

In conclusion, we first studied the correlation between m^6^A-related genes and the prognosis of LUAD, revealing that most of these genes had differential expressions between tumor and non-tumor tissues. Then, we screened out six genes directly related to prognosis via a comprehensive analysis to form a risk signature that could be used as an independent prognostic factor. This suggests that m^6^A-related genes (especially HNRNPC, METTL3, YTHDC2, KIAA1429, ALKBH5, and YTHDF1) may play an essential role in the occurrence and development of LUAD. Based on our study, it may not be appropriate and beneficial to inhibit or induce some of the m^6^A-related genes simply. Our study suggests that m^6^A-related genes may affect cancer development through particular patterns. Revealing these specific patterns can help the clinicians identify the high-risk types. Besides, molecular mechanisms play an important role in the relationship between the process of m^6^A and LUAD. Further investigations will provide more information on internal mechanisms. More prospective studies should be conducted to validate the prognostic function.

## Data Availability

The datasets supporting the conclusions of this article are available in TCGA database and the GEO database.

## Abbreviations

ALKBH5: alkB homolog 5
CDK1: Cyclin-dependent kinase 1
FTO: Fat mass and obesity-associated
HR: Hazard ratios
HNRNPC: Heterogeneous nuclear ribonucleoprotein C
LASSO: Least Absolute Shrinkage and Selection Operator
LUAD: Lung adenocarcinoma
m^6^A: N^6^-methyladenosine
mRNA: Messenger RNA
METTL3: Methyltransferase-like 3
METTL14: Methyltransferase-like 4
NSCLCs: Non-small-cell lung carcinomas
OS: Overall survival
PCA: Principal Components Analysis
RBM15: RNA-binding motif protein 15
rRNA: Ribosomal RNA
snRNA: Small nuclear RN
tRNA: Transfer RNA
uPAR: Urokinase receptor
VIRMA: vir-like m^6^A methyltransferase-associated protein
WTAP: Wilms tumor 1-associated protein
ZC3H13: zinc finger CCCH domain-containing protein 13.

## Supplementary Materials

Supplementary Materials Figures 1(a) and 1(b): the LASSO regression showed that the best lambda value was 6, indicating that a risk scoring signature contains 6 variables.
