# Supplementary material for: Deciphering N6-Methyladenosine-Related Genes Signature to Predict Survival in Lung Adenocarcinoma
Source: Biomed Res Int. 2020 Feb 29;2020:2514230. doi: 10.1155/2020/2514230 (PMC7066421; doi:10.1155/2020/2514230)
Supplement: Supplementary Materials — Figures 1(a) and 1(b): the LASSO regression showed that the best lambda value was 6, indicating that a risk scoring signature contains 6 variables. [file 2514230.f1.pdf]

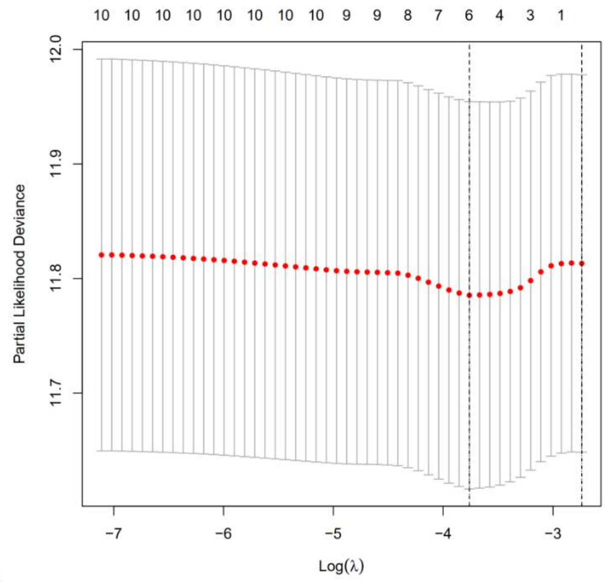

A

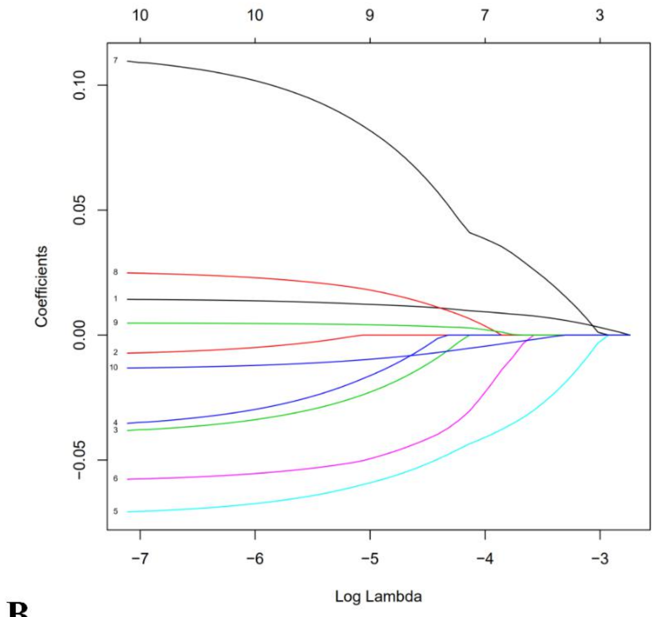

B

Figure 1 supplement. (A) (B) LASSO regression shows that the best lambda value is 6, which indicating a risk scoring signature contains 6 variables.
